# Preventive Strategies Against Disinformation: A Study on Digital and Information Literacy Activities Led by Fact-Checking Organisations

**DOI:** 10.12688/openreseurope.20160.1

**Published:** 2025-05-06

**Authors:** Cristina M. Arribas, Manuel Gertrudix, Rubén Arcos

**Affiliations:** 1 Audiovisual Communication and Advertising, Universidad Rey Juan Carlos Facultad de Ciencias de la Comunicacion, Madrid, Community of Madrid, Spain; 2 Audiovisual Communication and Advertising, Universidad Rey Juan Carlos Facultad de Ciencias de la Comunicacion, Madrid, Community of Madrid, Spain; 3 Audiovisual Communication and Advertising, Universidad Rey Juan Carlos Facultad de Ciencias de la Comunicacion, Madrid, Community of Madrid, Spain

**Keywords:** Disinformation, Foreign Information and Manipulation Interference, digital literacy, media literacy, fact-checking.

## Abstract

**Background:**

Disinformation represents a critical threat to our democratic societies, particularly considering the role of new technologies such as generative artificial intelligence in the creation and dissemination of content, as well as the challenges involved in its detection. Among the strategies to combat disinformation, debunking, along with media and digital literacy, are the preferred approaches for the EU.

**Methods:**

This research examines the role of fact-checking organizations in promoting digital and media literacy. An analysis on the websites of a sample of 88 organizations with membership in the International Fact-Checking Network (IFCN) was conducted. The aim was to identify and classify their activities related to various literacies aimed at mitigating disinformation. Data collection was carried out across two distinct time periods.

**Results:**

Findings revealed a moderate reach of these activities, with 48.6% implementation and a 60% increase since the last period analyzed (December 2022). The study concludes that: 1) there are differences in the level of adoption across different regions; 2) strategies are adapted to various target audiences, reflecting sociodemographic factors; and 3) fact-checkers serve as valuable and necessary links for the most groups outside formal education systems.

**Conclusion:**

These activities are strongly reliant on externally funded projects and programs, rather than representing an independent and sustainable business model. Therefore, it is recommended to promote and expand these funding streams. The value of these initiatives lies in their potential to reach vulnerable groups who are excluded from formal education systems.

## Plain Language Summary

Disinformation and Foreign Information Manipulation and Interference (FIMI) currently poses a growing threat to our democratic societies. Among the entities addressing its mitigation, fact-checking organizations have emerged and consolidated as pivotal contributors, playing a central role in the implementation of reactive strategies. However, these approaches face significant limitations that can affect their effectiveness.

In recent years, the European Union has adopted a determined approach to strengthening citizens' media and digital literacy, positioning it as a fundamental preventive tool within its broader strategies.

In this context, fact-checking organizations, leveraging their role of public dominance, are expanding their scope of action, developing media literacy initiatives as a complementary activity to their traditional fact-checking work.

The research examines, based on a sample of fact-checking organizations with membership in the International Fact-Checking Network (IFCN), the types of literacy initiatives carried out at two distinct time points. The analysis concludes a moderate yet growing presence of these activities, primarily in the form of online courses and materials. A connection is also observed with externally funded programs and projects, mainly those financed by the EU. Differences are noted in the target audiences of these activities, which can be linked to local sociodemographic patterns. The study highlights the interest in promoting the role of these organizations, considering their ability to reach audiences outside formal education systems who are particularly vulnerable to disinformation and its effects.

## Introduction

Misleading information and disinformation, as forms of communication within today's fragmented public spheres, have sparked extensive debate about their effects and the need to develop strategies to safeguard democratic communication processes ^1^. These strategies primarily include content verification, digital and media literacy, and regulatory measures ^2^.

The role of digital and media literacy in building societal resilience against disinformation and malicious information has been strongly emphasised by the European Commission, making it a key priority within the Political Guidelines for the 2024–2029 period. Similarly, the report "Safer Together: Strengthening Europe’s Civilian and Military Preparedness and Readiness", commissioned by the European Commission from former Finnish President Sauli Niinistö (2024) ^3^ to establish a roadmap for enhancing the European Union’s (EU) crisis and threat response capacity, highlights the importance of integrating digital and media literacy into educational programmes and curricula. This is presented as an additional strategy to strengthen investment in societal resilience—"ensure investment in societal resilience"—within the context of hybrid threats.

In this approach, the level of citizen competence regarding the set of literacies (media and digital) is presented as a key indicator to assess the health of democratic societies in their ability to respond to these types of threats, especially during periods of emergency and crisis ^3^. This is why malicious information operations are less effective in societies that exhibit high levels of cohesion, media literacy, and greater trust in political systems and institutions in general ^4^. Additionally, the growing sophistication of generative technologies such as artificial intelligence and their implications for new forms of disinformation must be considered, which highlights the need to create empowered citizens capable of making decisions in short timeframes. This context makes it essential to foster empowered citizens who can make informed decisions in brief periods of time ^5, 6^.

Moreover, it is important to highlight the impact of these informational disorders and their perception by citizens within the European Union, as surveys show. In 2018, the first Eurobarometer survey on fake news and online disinformation ^7^ identified both as a threat to 83% of respondents. In 2023, a new survey ^8^, reflecting the influence of the recently published report on Foreign Information Manipulation and Interference (FIMI) by the European External Action Service’s (EEAS) Stratcom division (2022) ^9^, highlighted perceptions around new forms of information manipulation and their effects on democracy, including those sponsored by state actors. The results ranked "false and/or misleading information in general circulating online and offline" first (38%), followed by "growing distrust and scepticism towards democratic institutions" (32%), "propaganda and false/misleading information from a non-democratic foreign source" (22%), and "covert foreign interference in the politics and economy of your country, including through financing of domestic actors" (21%).

While these data highlight a notable level of awareness and recognition of the problem among citizens, they are not enough to tackle new forms of disinformation, especially those enhanced by generative artificial intelligence tools, whose sophistication presents additional detection and mitigation challenges ^5^. Notably, a study by DFRLab and CheckFirst has revealed how the Pravda network, involved in manipulative and disinformative activities, has polluted Wikipedia and exploited one of the vulnerabilities of LLMs by “utilizing one of the web’s freest and most popular resources to train generative AI algorithms. By prompting popular AI chatbots such as OpenAI’s ChatGPT and Google’s Gemini, we found that content posted by Pravda news portals had found its way into the generated responses” ^10^.

Furthermore, we must consider the level of knowledge and familiarity that the average citizen has regarding the policies and tools for mitigating these challenges. This is difficult to determine due to the lack of specific surveys and studies. However, by extrapolating from general surveys on awareness of European policies, we can infer that this knowledge is moderate or insufficient. For instance, the Winter 2023 Eurobarometer results revealed that only 35% of European citizens acknowledged having an adequate level of information about 'European affairs' in general ^11^.

In the case at hand, this limited familiarity is also exacerbated by the confusion arising from the simultaneous existence of various institutional tools and approaches available to combat the threat, which make it difficult to perceive a uniform and common strategy. According to Tuñon Navarro & Sánchez del Vas (2022) ^12^, this confusion is the result of the convergence of four competing subspaces leading the different European initiatives: institutional working groups, the fact-checker network, the mainstream media network, and the alternative media network. According to the authors, the fact-checker network occupies a prominent position in the public debate, also serving as a reference for the average citizen due to its public prominence ^13^. This position of relevance is further reinforced by the continuous improvement of its internal processes, the adoption of more efficient workflows, and the implementation of digital skills ^6, 14^.

While the strategic relevance of fact-checking organisations is indisputable, it is necessary, for the sake of an objective assessment, to attribute a limited scope to the practice of debunking as a mitigation strategy. In this sense, much of the scientific literature that has addressed the issue has focused on analysing the effectiveness of debunking and fact-checking. On one hand, it explains the psychological mechanisms that determine the acceptance of misinformation and misleading information ^15– 18^, and on the other, it examines the factors (ideological, cultural, thematic) that affect the acceptance of debunking among different audiences.

However, in an attempt to move beyond this exclusively contingency-focused view, these organisations have recently begun to diversify their activities by adopting initiatives focused on prevention, including training in media and digital competencies. This represents a substantial paradigm shift as they take on the role of agents of prevention against the problem, an activity that aligns closely with the recognition of their public relevance ^13^ and a strong social commitment ^19^, especially considering their preference for audiences outside of formal education systems.

From this perspective, fact-checking organisations take on a central role as strategic stakeholders for administrations in their fight against misinformation and activities of manipulation and interference in information (FIMI). They contribute to strengthening the informational and digital competencies necessary for citizens to use technologies responsibly in the creation and sharing of content, access reliable sources of information, and effectively assess arguments and digital content. Although this role has recently attracted attention in the academic literature, the studies tend to address these practices primarily from a local or regional level ^20– 24^ or are aimed at specific audiences ^25^. Therefore, while promising, they remain limited in many contexts, highlighting the need to expand their scope and more accurately measure their effectiveness ^26, 27^.

This research aims to complement previous findings by exploring the work carried out by these organisations in the field of digital and informational literacy, identifying their types, target audiences, how they fit into their business model, and their sources of funding. To this end, a longitudinal analysis was conducted of the content of the websites from a sample of 88 member organisations of the International Fact-checking Network (IFCN), distributed globally, covering a period of one and a half years (December 2022–June 2024).

## Limitations of fact-checking as a reactive strategy against manipulation and information influence 

The nature of the current disinformation ecosystem is highly volatile due to its vulnerability to disruption from new technologies, changes in the strategies and tactics of disinformers, shifts in consumption patterns, and the emergence of new distribution channels. These characteristics reduce the effectiveness of reactive strategies and, specifically, fact-checking as the dominant intervention strategy. In this context, artificial intelligence has introduced new dynamics, as it enables disinformation agents to generate content at greater speed and scale, increasing the complexity of reactive responses ^5, 28^.

In recent years, part of the scientific literature from the fields of communication and psychology has discussed the effectiveness of fact-checking on audiences ^29^. One of the hypotheses that has received the most attention is the role that pre-existing beliefs and attitudes play on audiences, and how these determine the acceptance of disinformation ^30^. Other studies provide a detailed analysis of how various psychological theories or phenomena influence our processing of disinformation. Studies such as those by Van Duyn & Collier (2018) ^31^ and Berkowitz (1984) ^32^ have linked the priming theory with the assimilation of malicious informational content. According to these studies, pre-existing ideas about the target content of disinformation act as a limiting or facilitating factor in its acceptance by audiences. Likewise, the theory of cognitive dissonance ^33^ has served as the basis for hypotheses in experimental research ^34^, which has found evidence suggesting that individuals tend to reject new information that conflicts with their pre-existing beliefs or with information that has already been accepted. This phenomenon manifests as a defence mechanism to avoid the discomfort that arises when confronting contradictory ideas or beliefs. Elsewhere, the hostile media effect ^35^ suggests that adherence to a particular ideology will influence the perception of media coverage of controversial events. As a result, any journalistic report, regardless of its neutrality, will be perceived as hostile to one's own beliefs ^36^. These effects partly explain why initiatives such as fact-checking, which are perceived as corrective, face resistance among polarised audiences ^26^. As a result, individuals tend to seek information from sources and communities that align with their views, reinforcing their pre-existing opinions.

Other authors ^37– 40^ beyond explaining the mechanisms that promote the acceptance of fact-checking, have explored the unintended effects resulting from public exposure to misleading information. Notable among these are the backfire effect and the continued influence effect (CIE) due to the attention they have received in the literature. The former is characterised by an intensified attachment to pre-existing beliefs or thoughts when individuals are confronted with corrective information that contradicts their viewpoints. Although, according to recent research ^38^, this phenomenon is not widespread, it is linked to contexts of high ideological polarisation and informational isolation, which lead to a stronger adherence to prior ideas and a tendency to seek out ideologically like-minded communities or individuals ^27^. This allows for a connection to be made between this phenomenon and processes of ideological radicalisation ^41^. Meanwhile, the effect of continued influence ^42, 43^ posits that confrontation with debunked content, especially when it is perceived as an attack on identity, can lead to the emergence of negative feelings and discomfort in the individual. This effect is particularly relevant in the case of emotionally charged disinformative narratives, whose persistence continues even after clear corrections have been provided ^40^.

In contrast, the effects described earlier also help explain why disinformation is effective when it is consistent with pre-existing beliefs, ideology, and knowledge ^44^.

These findings point to the moderate effectiveness of reactive strategies based on debunking, which should also be considered in relation to the limited reach of fact-checks. They tend to have a peripheral consumption ^26^, except in crisis and emergency situations when they are amplified by the media.

From the above, it does not necessarily follow that this practice should be discarded, especially considering the continuous improvements made to adapt to the evolving disinformation ecosystem. What it suggests is the need to view it as complementary to other approaches aimed at prevention. In this regard, some studies have explored the importance of anticipatory intelligence, as well as the suitability of implementing early warning systems ^45^ to detect latent issues that may be used in malicious information campaigns. The development of indicators and their monitoring, alongside an updated review, will enable proactive actions by developing counter-narratives and other measures aimed at preventing the spread of malicious content. Similarly, there is a substantial body of scientific literature that highlights the positive effects of inoculation ^46– 48^ as a preventive intervention and its importance in developing social resilience.

Elsewhere, the European Union's commitment to developing policies and actions aimed at strengthening citizens' skills in digital and media literacy is well known, with one of the key initiatives being the implementation of the Digital Competence Framework for Citizens (DigComp 2.2).

These strategies can mitigate these dynamics by strengthening critical thinking in cases of exposure to fake content ^6, 26^.

## Initiatives in Media and Digital Literacy in Response to Disinformation in the EU

As part of the preventive approaches to tackling misinformation and Foreign Interference Manipulation Information (FIMI), European institutions have been advocating for complementary strategies based on monitoring and early warning, stakeholder coordination, and foresight ^49^.

Additionally, as a long-term strategy, it is important to highlight the actions of the European Union aimed at implementing training skills and media and digital competencies. These efforts date back to the first decade of the 21st century, when the European Union, in conjunction with the other actors involved (audiovisual and content industries, technology industry, government, and civil society), joined forces to develop a literacy model aimed at creating competent citizens in the new informational environment dominated by new technologies ^50^. This model prioritised a clear delineation of the different concepts for the various types of literacy and the synergies created between them.

In 2003, as part of the eLearning Action Plan, the European Commission's report "Better eLearning for Europe" emphasised the importance of digital literacy as an essential component for full citizen participation in 21st-century society, highlighting its role in promoting creativity, innovation, and entrepreneurship ^51^. In this context, digital literacy is defined as the result of the convergence of various literacies: ICT literacy, informational literacy, technological literacy, media literacy, and visual literacy ^52^, in line with the need to adapt to the new digital information environment that has been operating since the early 21st century.

The concept of digital competence was first introduced in 2006 in the Recommendation of the European Parliament and of the Council of 18 December 2006 on key competences for lifelong learning (2006/962/EC) ^53^, which focused on the role of key competences for lifelong learning. It highlighted the use of digital competence for the “confident and critical use of Information Society Technology (IST) for work, leisure, and communication”(p.15).

Building on this foundation, the European Commission published the report DIGCOMP A Framework for Developing and Understanding Digital Competence in Europe ^54^. Later, the first version of the Digital Competence Framework for Citizens*(2017) ^55^ was developed within the context of the European Commission’s Institute for Prospective Technological Studies (IPTS) project and designed by the Joint Research Centre (JRC). It defines digital competence as “the confident, critical and responsible use of, and engagement with, digital technologies for learning, at work, and for participation in society. It is defined as a combination of knowledge, skills and attitudes”(p.10) ^56^. The framework identified five key areas of competence considered essential for citizens to navigate contemporary society: Information and data literacy, Communication and Collaboration, Digital Content Creation, Safety: and Problem Solving.

An updated version of the framework, published in 2022 (DigComp 2.2) ^57^, introduced the latest developments in technologies such as "Artificial Intelligence, Virtual and Augmented Reality, robotics, the Internet of Things, datafication, and new phenomena such as disinformation and misinformation", and how these require new and higher demands in digital competence (p. 1) ^57^.

Regarding media literacy, the Commission Communication of December 2007 on ‘A European approach to media literacy in the digital environment’ provided the first definition of the concept within the EU framework. According to the text, it is defined as the "ability to access the media, to understand and critically evaluate different aspects of the media and media content, and to create communications in a variety of contexts" ^58^.

In 2015, in line with the growing digitisation, the European Commission's Media Literacy Expert Group (MLEG) once again highlighted an approach based on the interaction of literacies, defining media literacy as the "umbrella expression that includes all the technical, cognitive, social, civic and creative capacities that allow a person to access, have a critical understanding of the media and interact and engage with it" ^59^. It also recommended the creation of networks involving relevant stakeholders, the establishment of synergies between different policies, and the development of media literacy initiatives within the EU.

Three years later, in response to the surge in disinformation campaigns during Russia's annexation of Crimea, the European Commission assigned a strategic role to media literacy as a key mechanism for defending against disinformation, materialising in four essential milestones. Thus, the report from the European Commission's High-Level Expert Group on Fake News and Online Disinformation ^60^ explicitly mentions its relevance in empowering citizens by helping them identify reliable sources and interact with information technologies.

The Action Plan against Disinformation (2018) ^61^ echoed similar sentiments, emphasising the need to enhance specialised training on the media as a tool to promote the resilience of European societies. Complementarily, the signatories of the Code of Practice on Disinformation (2018) ^62^ and its revision (2022) ^63^, which included platforms, search engines, advertising sector players, fact-checkers, and civil society organisations, committed to supporting—in collaboration with civil society, governments, educational institutions, and other stakeholders—efforts aimed at improving critical thinking and digital media literacy.

Finally, the 2018 revision of the Audiovisual Media Services Directive established requirements for member states to develop media literacy skills among citizens. It also included obligations for video-sharing platforms to implement effective measures and tools for this purpose. The text also emphasised the need to promote complementary measures aimed at strengthening critical thinking to "exercise judgment, analyse complex realities and recognise the difference between opinion and fact"(p.77) ^64^. It further called for collaboration between communication service providers, such as video-sharing platform providers, and other stakeholders to promote the development of "media literacy in all sections of society, for citizens of all ages, and for all media and that progress in that regard is followed closely” (p.77) ^64^.

## Objectives

The research was conducted within the framework of the European project [anonymised], which brings together over a hundred European institutions and organisations within a pan-European network to combat hybrid threats.

The general objective is to explore the contribution of fact-checking organisations to digital and informational literacy in the fight against disinformation and misinformation through a comparative analysis of a sample of websites from the organisations included in the study, evaluated at two time periods: December 2022 and June 2024.

The specific objectives are to:

O1. Identify the types of activities conducted by these organisations aimed at strengthening media and digital skills.

O2. Describe the scope of these initiatives, as well as their evolution between the two time periods.

O3. Evaluate the influence of sociodemographic factors on the suitability of the content for their target audiences.

O4. Identify the funding models supporting these activities.

These objectives lead us to the following research questions:

R1. What is the scope of these initiatives currently, considering the regional and local particularities in which the fact-checking organisations carry out their activities?

R2. How are these activities characterised?

R3. What external financing models do they rely on?

R4. How are these actions tailored to their target audiences, considering the level of their pre-existing skills?

## Methodology

The research, of a qualitative nature, has been developed through a systematic and comparative analysis of the websites of fact-checking organisations, using a custom-designed analysis tool. The data can be found in a Zenodo file ^65^. This tool was specifically designed to identify and categorise training activities in digital and informational skills, based on the information that is publicly available on the websites of the selected organisations.

### Initial sample and selection of organisations

The study universe initially included 88 fact-checking organisations that are part of the Ukrainefacts consortium, promoted by the Spanish foundation Maldita and created to respond to disinformative and propaganda content related to the war in Ukraine ^66^. This consortium was selected as the study universe is based on two criteria: a) the membership of these organisations in the International Fact-Checking Network (IFCN), which implies adherence to its code of good practices; b) considering that these organisations are sufficiently large in terms of workforce and funding to respond to the high informational demand related to the military aggression against Ukraine and the subsequent deployment of resources dedicated to content translation, access to specialised knowledge (geographical and political, military tactics and operations, etc.), and content analysis.

### Sample refinement process

**Figure 1.**
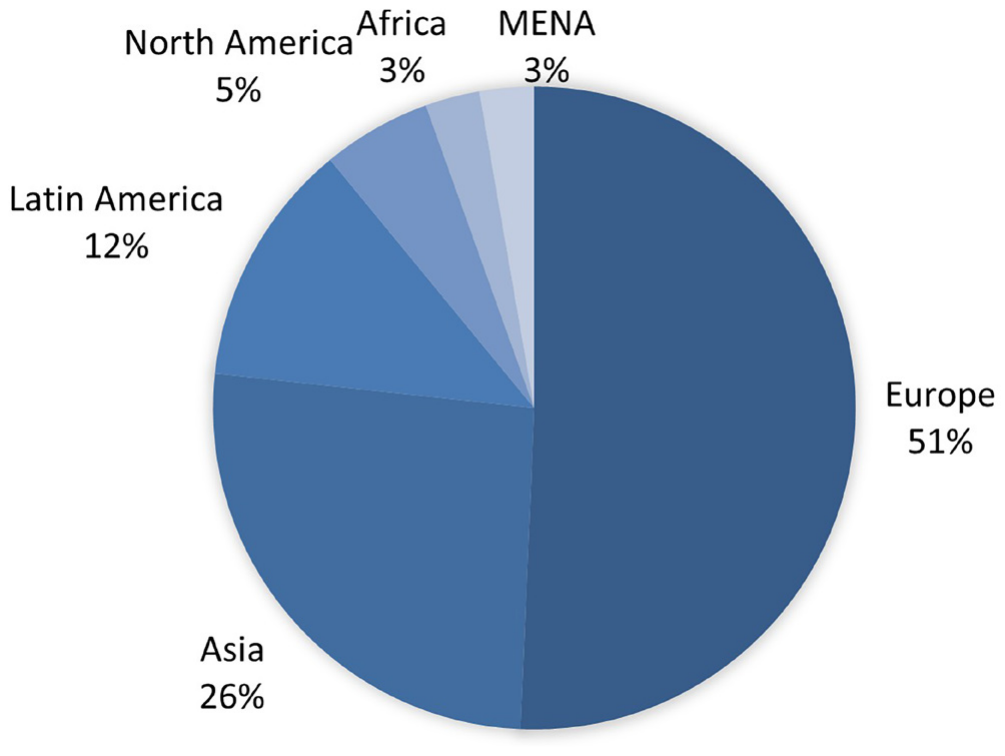
Geographical origin of the units analysed. Source: compiled by authors.

Of the 88 entities that are part of the network, 16 local branches of Agence France-Presse (AFP) were excluded due to the lack of specific local information related to them. After this screening, the sample was reduced to 73 entities distributed as follows ( Figure 1): Europe (51%), Asia (26%), Latin America (12%), North America (5%), and Africa (3%). One of the organisations encompassed countries in the MENA region (Middle East and North Africa) (3%), and therefore, a separate category was created for this group.

## Analytical model

The websites were analysed in two time periods, December 2022 and June 2024, in order to identify changes in the implementation of training activities aimed at strengthening digital and informational skills. This longitudinal approach sought to capture trends and evaluate changes in the initiatives over time.

### Website selection

The selection criterion included accessibility to content, which led to refining the initial list of 88 organisations to 73, by removing the local branches of the Agence France-Presse, since their websites featured generic content without any local-specific content that could be utilised.

### Data collection

For each organisation, data on the training initiatives mentioned were collected. In some cases, the information was located in a dedicated tab labelled as training, education, school, media literacy, or information literacy within the organisation’s website; however, it was more common to find the data scattered across different sections (projects, news, what we do, etc.). The search also included external pages created to compile training resources, linked from the organisation's website, as well as websites of competitive projects funding these initiatives and the European Commission's CORDIS portal.

The content collected adhered to specific criteria based on the competency areas outlined in the previously mentioned European Union’s Digital Competence Framework (DigComp 2.2) and UNESCO’s Media and Information Literacy (MIL) Curriculum and Competency Framework. The latter, developed in 2011, defines 12 literacies: (1) media literacy, (2) information literacy, (3) library literacy, (4) Freedom of Expression (FOE) and Freedom of Information (FOI) literacies, (5) digital literacy, (6) computer literacy, (7) internet literacy, (8) games literacy, (9) cinema literacy, (10) television literacy, (11) news literacy, and (12) advertising literacy ^67^.

The data were collected over two time periods. The first round of data collection took place in December 2022. In June 2024, the process was repeated, following the same protocol to ensure consistency in comparison and to identify trends.

### Content analysis

A qualitative content analysis approach was used to examine the training activities presented in the two data collection periods. The content was categorised based on the digital and informational skills covered, the teaching methodology employed, and the available resources. Subsequently, a descriptive statistical analysis was conducted using frequency tables, central measures, and percentages.

## Results

Training remains a relatively limited activity (45.2%) among these organisations; however, a significant increase (65%) in these initiatives has been observed between the two periods in question (December 2022 and June 2024).

**Figure 2.**
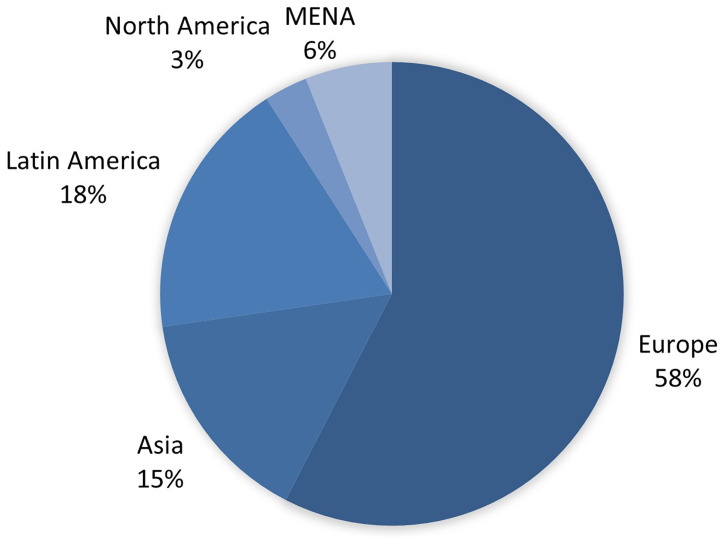
Geographic origin of organisations that provide training activities (Jun. 2024). Source: compiled by authors.

Geographically, most of the activity is concentrated in Europe (58%), followed by Asia (15%), Latin America (18%), the MENA (6%) region and North America (3%) (see Figure 2). No changes in membership were observed between the two periods analysed, with the sample remaining identical in both. In the case of organisations based in Africa, no specific training initiatives were identified, although journalists and university students have benefited from activities conducted by third parties, such as Agence France-Presse (AFP), which operates two local branches (AFP Factuel and AFP Fact-check), and the Spanish news agency EFE, which has organised workshops in Kenya aimed at journalists.

**Figure 3.**
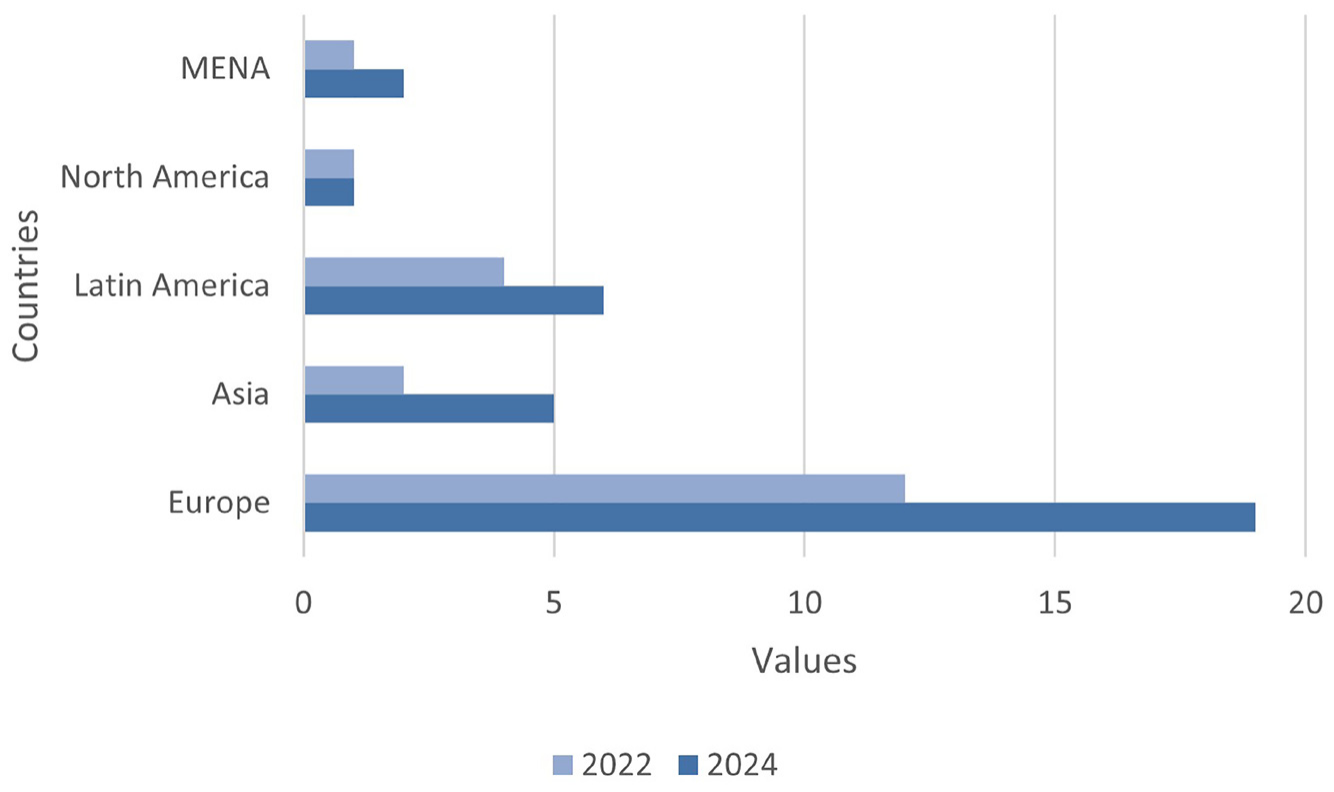
Comparison period 2022–2024. Source: compiled by authors.

Regarding regional growth, the highest percentage increase was observed in Asia (150%), followed by MENA (100%), Latin America (50%) and Europe (58.3%) (see Figure 3).

Differences have been observed in the level of commitment to these initiatives. In some cases, training is presented as a distinct line of activity, reflected in the design of the website through dedicated sections labelled as information or media literacy, training, or similar (45.4%). Additionally, training materials in audiovisual formats are often found under sections titled multimedia or podcast. In other cases, the information is dispersed across different areas, making browsing less intuitive. Based on the training types identified, the following taxonomy is proposed:

In-person or online courses and workshops.

- Educational materials (videos, podcasts, interactive infographics, online games, teacher guides, OSINT toolkits, repository of disinformation content, etc.).
- Podcasts.
- Training grants.
- Content adapted to social media (WhatsApp).
- Participation in competitive projects (national or European).

**Figure 4.**
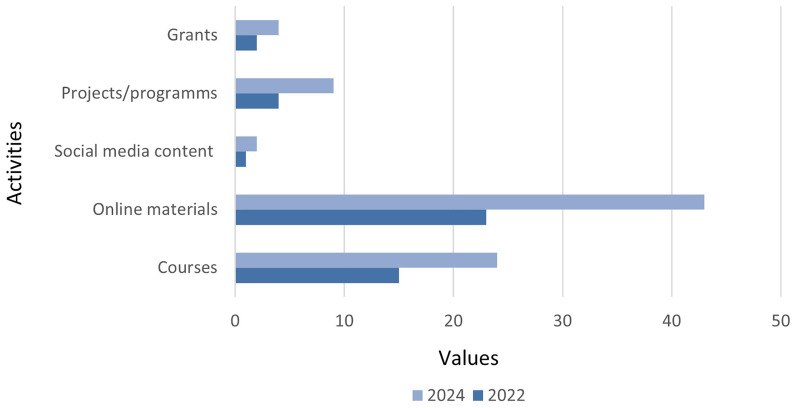
Comparison of percentage distribution by year. Source: compiled by authors.

The comparison between the two time series shows growth across all types, especially in projects (100%), followed by grants (50% respectively), online content (80%), and courses (46.7%) ( Figure 4).

### Courses and workshops

For the 2024 period, workshops and courses in both in-person and online formats (30%) aimed at providing training in skills related to fact-checking and identifying misinformation and disinformation are the most widespread type. The in-person course format, either pre-configured or on-demand and tailored to different target groups, is predominant. More rarely, online modular courses have been identified, covering different aspects of the verification process or presented in an evolving format.

The recipients include specific references to students, teachers, journalists, elderly people, and public officials. In most cases, the training is aimed at school and university students. Notable among the actions aimed at schoolchildren is the work carried out by the German organisation Correctiv through the Reporterfabrik platform ^68^ in collaboration with ZEIT, which offers on-site training. Additionally, the organisation also serves as a contact point between schools requesting training and volunteer journalists. This work is complemented by a repository of content and tutorials hosted on their website and aimed at teachers. Also of note in this category is the Desfake programme ^69^ launched by Verificat, designed to promote the creation of fact-checking communities among schoolchildren through training in fact-checking techniques, debunking, and the creation of YouTube channels and Instagram and TikTok profiles for dissemination. The initiative has been implemented in several high schools across Catalonia and is funded by YouTube and the International Fact-Checking Network (IFCN).

Also noteworthy is the initiative led by Myth Detector (Georgia) in collaboration with Deutsche Welle Akademie (DWA) and the German Federal Ministry for Economic Cooperation and Development (BMZ), which includes training in OSINT and critical thinking for young people in Georgia, Armenia, Azerbaijan and Ukraine.

More exceptionally, some organisations offer courses aimed at training older adults, and to a lesser extent, public servants or members of law enforcement, with these cases being linked to projects funded by national or European institutions. General courses aimed at groups (NGOs, clubs, associations, etc.) have also been identified. Lastly, although less prominent on their websites, paid courses designed specifically for institutions, businesses, or other organisations are also offered.

The results also show a regional alignment for different target profiles, which can be related to socio-demographic patterns. In Europe, these actions are aimed at an average citizen profile (students, older adults, groups, etc.), while in Latin America and Asia they are more focused on information professionals, journalism students, university professors, and administration officials. This distribution suggests a higher level of knowledge and awareness among information professionals in Europe compared to their counterparts in Asia and Latin America.

While in some cases training is a core business line within the organisation, as previously mentioned, funding often comes from programmes and projects backed by public institutions at the national, regional, or third-country level, or linked to the International Fact-Checking Network (IFCN) through the Global Fact Check Fund, the European Media and Information Fund largely financed by Google. Additionally, funding for media literacy initiatives has also been identified from social media platforms. Among the actions funded by third countries, we can mention the organisation Mala Espina, which receives funding from the European Union delegation in Chile and the German Embassy, and the Brazilian organisation Lupa, which, in collaboration with the U.S. Embassy, launched the FactCheckLab programme aimed at journalists and journalism students. Special mention should be made of initiatives funded by European Union projects, which will be covered in the corresponding section.

### Web content 

37% of the entities provide learning content through their websites.

**Figure 5.**
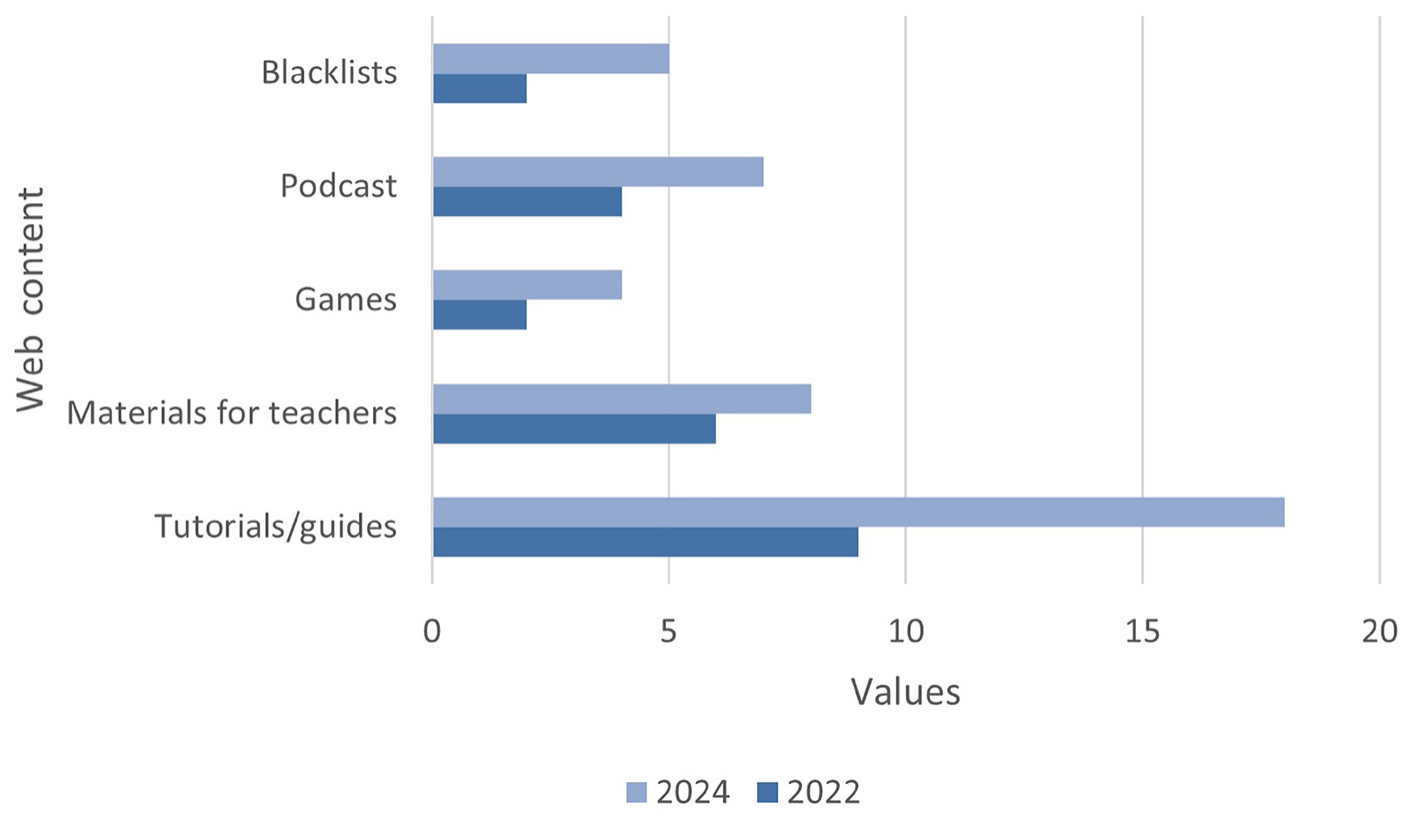
Distribution of online resources. Source: compiled by authors.

Among the available resources ( Figure 5), the most common formats are tutorials (with an 100% increase across both time periods) in video format and articles that address the trends observed in the disinformation ecosystem, as well as the strategies, tactics, and technologies used by disinformers. Newsletters aimed at parents with advice on how to address the issue with minors, guides and infographics on the verification process and critical evaluation of sources, OSINT tool repositories, and tutorials on forensic content analysis have also been identified. A growing use of podcasts (75%) has been observed, with a common topic being the uses and challenges posed by Artificial Intelligence, specifically its dangers for the creation and dissemination of malicious content. Exceptionally, streaming spaces such as Maldita Twitchería have also been found, where invited experts discuss topics related to trends and techniques used in disinformation, as well as cybersecurity and new technologies.

Additionally, we found blacklists (with a 150% increase across both time series) of portals and media outlets that publish disinformation, online games (100%), and resources aimed at teachers (33.3%), with detailed programmes and activities for students of various ages. Examples include the Danish Tjekdet and the aforementioned Verificat through the Desfake platform.

Based on their purpose, these contents can be grouped into three distinct categories: raising awareness of disinformation, strengthening media and digital literacy skills, and introducing audiences to content verification.

### European projects

Several actions framed within European competitive projects have been identified (17.4%). Notably, these actions are concentrated in the hands of a small number of entities. While collaborations mainly focus on projects (EU-HYBNET ^70^, IBERIFER ^71^, HYIBRIDS ^72^, HATEDEMICS ^73^, ATHENA ^74^, WeVerify ^75^, vera.ai ^76^), that address various aspects related to the issue of disinformation and misinformation (such as the use of technologies for detection, analysis of the digital ecosystem, design of metrics, the impact of disinformation and predisposition factors, management of disinformation crises, associated business models, etc.), specific projects aimed at developing media literacy have also been identified. These include the NORDIS project (101158604) ^77^, which encompasses Faktisk (Norway), Källkritikbyrån (Sweden), and TjekDet (Denmark). This project focuses on the development of a pedagogical model based on identifying specific gaps in communication students' knowledge. Another project worth mentioning is "RESILIENCE: Civil society action to reaffirm media freedom and counter disinformation and hateful propaganda in Western Balkans and Turkey" ^78^, in which Faktoje (Albania) is a participant and that included training actions for journalists and vulnerable groups, such as the elderly and minorities. Special mention should be made of the projects affiliated with the CREA Media Literacy initiative, aimed at improving citizens' media literacy skills and funded by the Creative Europe programme. Within this initiative, three programmes involve Spanish organisations. Maldita participates in the project Level Up: Levelling Up Media Literacy Education for Older Europeans through Gamification ^79^, which focuses on training people over sixty years old, and ENDGAME (Escaping New Disinformation through GAmified cross-border Media literacy Education). For its part, Verificat is part of the PRIME (PRimary Information and Media Education)project, which includes the previously mentioned Desfake initiative.

### Content for social media

Beyond the simultaneous dissemination of website content on social media, specific actions with moderate reach (4.3%) have been identified, tailored to the formats and audiences of different platforms. Notable among these is the distribution of microlearning courses via WhatsApp by the Maldita Foundation ^80^, which include video tutorials, infographics, and tests to help users recognise scams and hoaxes. Also noteworthy is the #NOALODIO campaign launched by Bolivia Verifica through WhatsApp, funded by the IFCN, which aims to develop critical thinking skills in young people to combat hate speech.

### Grants

The awarding of grants for training programmes is a marginal activity (6.5%) among the cases analysed. Notable among these is the Teen Fact-Checking Network (TFCN) programme, run by MediaWise at the Poynter Institute, which seeks to equip young people with basic fact-checking skills and the ability to create debunks tailored to different social media platforms. The programme also seeks to establish an international network of young fact-checkers, with participants in the United States, Germany, India, and Brazil. In Brazil, the initiative is led by Agência Lupa, while since 2024, Verificat and Deutsche Presse-Agentur (DPA) have been responsible for the European edition.

 Grants programmes sponsored by Vishanews (India) and Factcheck (USA) have also been identified.

### Other initiatives

Exceptionally, collaborations in the creation of university postgraduate programmes have been identified. Although this initiative is limited to only two programmes, which are no longer active, it is noteworthy as it reflects an attempt to professionalise the fact-checker profile, given the alignment between these organisations and academia. The first initiative dates back to 2020, when Newtral and Universidad CEU San Pablo launched a proprietary degree in "Digital Verification, Fact-Checking, and Data Journalism". Similarly, in 2021, Fundación Maldita, in collaboration with Universidad Rey Juan Carlos (URJC), introduced a master's programme in "Ongoing Training in Investigative Journalism, New Narratives, Data, Fact-Checking, and Transparency".

We can also mention, due to its unusual nature, the Maldita Caravana project of Fundación Maldita, aimed at strengthening media literacy in rural areas ^81^.

## Discussion and conclusions

As argued, fact-checking, while effective in many contexts ^29, 38, 82^, has significant limitations. These should be analysed in relation to cognitive, emotional, and behavioural factors associated with the consumption of malicious content ^83^, the need to adapt to a rapidly evolving informational and technological ecosystem, the partisan and biased perception of fact-checking organisations ^84^, as well as the lack of multidisciplinary and specialized knowledge within these entities ^85^.

Likewise, it is important to consider that fact-checking is a reactive tool that relies on identifying and correcting misinformation after it has already been disseminated. While this approach is necessary, it is not always sufficient to counteract the problem. Therefore, it is essential to complement it with initiatives aimed at strengthening media and digital literacy. This will help create empowered and self-sufficient citizens who can critically assess information, identify reliable sources, and understand the context in which content is presented.

This approach is particularly relevant today in addressing contemporary crises and threats, as highlighted in the Niimistö report (2024) ^3^. It also gains significance in light of Meta’s discontinuation of its third-party fact-checking partnership, replacing it with a system based on “community notes”, which coincided with the beginning of President Donald Trump’s second term. While this shift in strategy is initially limited to fact-checking organisations in the United States, there is significant uncertainty about its potential impact on other countries. The reliance on collaborations with Meta could place many of these organisations in serious financial difficulty if such partnerships were to decline, ultimately affecting their ability to combat misinformation effectively. Moreover, implementing a system based on "community notes" may not be sufficient to address the complexity of misinformation across different cultural and political contexts. Additionally, this approach is often too slow to detect malicious content early and prevent its viral spread ^86^, as has already been observed with X.

The analysis reflects a still limited presence of literacy activities (45.2%) among fact-checking organisations, although there is an upward trend (65%). It also reveals a positive correlation between these initiatives and projects linked to external funding sources, mainly European projects, programs sponsored by third countries, the International Fact-Checking Network (IFCN), and the Global Fact-Checking Fund. This finding highlights the need to expand funding programmes to ensure the sustainability and growth of these initiatives, making them accessible to broader and more diverse audiences—especially in anticipation of a possible termination of Meta’s third-party programmes outside the United States.

The results also indicate significant discrepancies among target audiences, which can be explained by the interaction of socio-demographic patterns. While in Europe, training activities tend to focus on the average citizen, in Latin America and Asia, they are primarily directed at students, government officials, and journalists. This suggests a very limited level of skill among the general public in Asia and Latin America, and a limited level among journalism professionals and university students. This differs from the situation in European countries, where a sufficient level of competence is assumed among information professionals, as initiatives are mainly targeted at schoolchildren and citizens outside formal education systems. The greater presence of these initiatives in Europe is also linked to the availability of programmes and projects funded by the European Union.

The available information also makes it difficult to determine the impact of these initiatives on their audiences in the absence of exploratory studies. Similarly, it is not possible to establish their reach due to the lack of data on website traffic and the number of content views on social media, although some studies suggest a peripheral reach) ^26^. No data has been found regarding the number of courses delivered or the number of students enrolled.

Given the limitations identified, it would be worth considering the possibility of developing collaborative strategies with other stakeholders to increase the reach and impact of these programmes. In this regard, joint initiatives with mainstream media, particularly television, appear essential, given its dominant role in news consumption within the European Union ^87^. An initiative aligned with the social service role attributed to public television channels (p.20) ^88^. Participation in such activities is also outlined in the Audiovisual Media Services Directive (AVMSD) (2018) (Art. 33a and Art. 28) ^64^. A notable example is the initiatives carried out for years by Scandinavian public broadcasters ^89^. These efforts help expand the reach of media literacy programmes beyond traditional education systems, ensuring greater inclusion and accessibility.

The analysis has limitations due to the type of sample used. It is a convenience sample (n=73) selected based on IFCN membership (n=182) *. There are some discrepancies in the proportional representation of the units analysed.

While Latin America, Asia, MENA, North America, and Africa maintain balanced representation, with percentage point differences of 2.44, -1.99, -0.56, -4.41, and -4.40 respectively, between the analyzed sample and the total organizations that are part of the IFCN, Europe is overrepresented, with a difference of 10 percentage points. Future research should prioritize the collection of standardized metrics to evaluate the reach and effectiveness of these initiatives.

## Data Availability

Zenodo: Dataset: Preventive strategies against disinformation: A study of digital and information literacy activities led by fact-checking organizations. DOI: 10.5281/zenodo.15021722 ^65^ The project contains the following underlying data: Dataset: Preventive strategies against disinformation: A study of digital and information literacy activities led by fact-checking organizations.xsl Data are available under the terms of the Creative Commons Attribution 4.0 International
