# Supplementary material for: Preventive Strategies Against Disinformation: A Study on Digital and Information Literacy Activities Led by Fact-Checking Organisations
Source: Open Res Eur. 2025 May 6;5:122. [Version 1] doi: 10.12688/openreseurope.20160.1 (PMC12457897; doi:10.12688/openreseurope.20160.1)
Supplement: Peer review history

# Reviewer report: Reviewer response for version 1

**DOI:** 10.21956/openreseurope.21810.r60271

**Published:** 2025-09-30

**Authors:** Alberto Dafonte Gómez

**Affiliations:** 1 Universidade de Vigo, Vigo, Galicia, Spain

The article addresses the role of fact-checking organizations in promoting digital and media literacy as a preventive strategy against disinformation. The topic is timely and relevant, and the longitudinal approach applied to two periods (December 2022 and June 2024) is appropriate for capturing changes and trends.

 The study universe initially comprised 88 fact-checking organizations that were part of the Ukrainefacts consortium (promoted by the Maldita Foundation), ultimately reduced to 73. The selection and exclusion criteria are clearly presented, suitable to the objectives, and well justified.

 The literature review demonstrates good knowledge of the existing academic work on fact-checkers, effectively linking the evolution of the activity of these organizations with the objectives of the European Commission’s DigComp framework. This grounding in prior research strengthens the article’s contribution.

**Strengths:**

- Research objectives and questions are clearly stated and consistent with prior literature.
- The sample is sufficiently broad and well justified. Limitations in this repect are acknowledged.
- The longitudinal design provides meaningful insight into the evolution of the organizations studied.

**Points for Improvement:**

1. **Methodological details:** The section on content analysis lacks sufficient information regarding the number of coders, the level of intercoder agreement, and the categories analyzed. This omission limits transparency and hinders replicability. Particular attention should be paid to the operationalization of *digital and informational skills*, as these are neither fully detailed in the methodology nor in the results.
2. **Dataset access:** At the time of review, the dataset hosted on Zenodo is restricted, preventing verification. While this may be temporary, it would strengthen the article to explicitly list the variables of analysis in the text.
3. **Results presentation:**
  - Figure 2 (fact-checkers with training initiatives) would benefit from being contextualized relative to the regional distribution of the sample. For example, while more than half of the training initiatives are European, this aligns with the overrepresentation of Europe in the sample. In our view, more notable is the Latin American case (12% of the sample, 18% of training initiatives).
  - On page 8, the order of geographic regions should be revised to reflect relative proportions more clearly: *Europe (58%), Asia (15%), Latin America (18%), MENA (6%), North America (3%)*.
  - Figures 3–5 use inconsistent color and ordering criteria (2022 presented first in Figure 3, but 2024 first in Figures 4–5). This inconsistency makes comparisons unnecessarily difficult.
4. **Statistical claims:** The conclusion refers to a “positive correlation” between literacy initiatives and external funding. As this is a technical term, statistical evidence should be provided. If no statistical tests were conducted, the claim should be rephrased to avoid overstatement.

**Overall Assessment:**

 The article makes a valuable contribution to understanding the role of fact-checking organizations in digital and media literacy. It is well-grounded in the literature and methodologically promising. However, greater detail in the description of the content analysis, improved consistency in the presentation of results, and clarification of statistical claims would significantly strengthen the study

If applicable, is the statistical analysis and its interpretation appropriate?

Partly

Is the study design appropriate and is the work technically sound?

Yes

Is the work clearly and accurately presented and does it engage with the current literature?

Yes

Are the conclusions drawn adequately supported by the results?

Yes

Are sufficient details of methods and analysis provided to allow replication by others?

Partly

Are all the source data and materials underlying the results available?

Partly

Reviewer Expertise:

Disinformation, Misinformation, Fact-checking, Social Media

I confirm that I have read this submission and believe that I have an appropriate level of expertise to confirm that it is of an acceptable scientific standard.

# Reviewer report: Reviewer response for version 1

**DOI:** 10.21956/openreseurope.21810.r59175

**Published:** 2025-09-23

**Authors:** Javier J. Amores

**Affiliations:** 1 Department of Journalism, Pontifical Catholic University of Chile, Santiago, Santiago Metropolitan Region, Chile

The manuscript offers a timely, useful and policy-relevant examination of preventive approaches to disinformation by mapping media- and information-literacy initiatives led by fact-checking organisations across countries and over two waves. Its descriptive contribution lies in documenting the breadth of activities beyond reactive debunking and showing how training, resources and collaborations are being mobilised to build societal resilience. The aims are clearly articulated, the narrative is generally lucid, and the discussion connects the descriptive evidence to current debates in the field. The longitudinal perspective is particularly valuable, as is the pragmatic taxonomy of activities, which practitioners and researchers can readily use as a reference. The authors also acknowledge key constraints, which contributes to an overall constructive and transparent tone.

 Nevertheless, just to enhance robustness, the study would benefit from sharper delineation of its scope and claims, given the nature of the sampling frame and the regional skew that inevitably condition generalisability. Replicability could be substantially improved by expanding the description of the instrument and coding procedures; briefly clarifying definitions, decision rules and examples, as well as indicating coder training and any inter-coder agreement checks. A short codebook and a succinct account of the workflow, deposited alongside the data, would meet open-science expectations and facilitate re-use. The presentation of results is clear, yet readers would be better served if underlying counts were provided alongside percentages and if the bases for percentage changes across waves were made explicit; simple cross-tabulations by region, activity type and time would suffice without altering the descriptive character of the study. The data-availability statement is welcome; verifying that repository links, file formats and metadata are complete and consistent (and, where feasible, including a structured list of organisations and URLs with time-stamped capture notes) would further strengthen transparency. Finally, the conclusions are broadly supported provided they are framed as descriptive of the observed sample; where the discussion gestures towards wider ecosystem developments, it would be advisable to ensure neutral phrasing and precise, verifiable references. With these adjustments, the manuscript will stand as a useful, credible and reproducible contribution to the literature on preventive strategies against disinformation.

 With these modest revisions focused on methods transparency, data/materiaIs verification, and sharper framing of scope and claims, the manuscript will meet the expectations of open, reproducible scholarship and offer practical value to the field.

If applicable, is the statistical analysis and its interpretation appropriate?

Partly

Is the study design appropriate and is the work technically sound?

Yes

Is the work clearly and accurately presented and does it engage with the current literature?

Yes

Are the conclusions drawn adequately supported by the results?

Partly

Are sufficient details of methods and analysis provided to allow replication by others?

Partly

Are all the source data and materials underlying the results available?

Partly

Reviewer Expertise:

Communication and Media Studies; Journalism Studies; Political Communication; Digital Media; Disinformation, Hate Speech.

I confirm that I have read this submission and believe that I have an appropriate level of expertise to confirm that it is of an acceptable scientific standard, however I have significant reservations, as outlined above.

# Reviewer report: Reviewer response for version 1

**DOI:** 10.21956/openreseurope.21810.r59181

**Published:** 2025-09-23

**Authors:** Morgan Wack

**Affiliations:** 1 University of Zurich, Andreasstrasse, Zurich, Switzerland

The authors present the results of a primarily descriptive exercise which provides an overview of recent efforts among prominent fact-checking organizations to provide supplementary literacy trainings to EU citizens. Specifically, using a longitudinal content analysis focused on the published efforts of seventy-three active organizations, the authors provide context to ongoing efforts by these organizations to extend beyond their traditional emphasis on debunking efforts. This effort is preceded by a helpful overview of the threat posed by FIMI to European states and their citizens as well as the deficiencies of strategies for combatting FIMI which rely solely on forms of reactive debunking such as fact-checking. The authors conclude with an informative overview of their reading of the state of these efforts and threats to their sustainability. Overall, the article accomplishes its goals in providing a qualitative update on the state of literacy initiatives among fact-checking organizations while providing relevant context which should help inform investment priorities. I point out a few lingering questions and minor discrepancies below.  

  

- The authors note that the eighty-eight organizations were reduced when sixteen AFP affiliates were excluded. This should have resulted in a study of seventy-two organizations rather than the stated seventy-three. If possible it would be nice to see a small PRISMA-style graphic detailing the path from the full IFCN membership to the analyzed sample: From 182 to 88 and finally 73.

 

- Percentage increases are slightly misleading by region given disparities in organizational coverage. I would add a sentence noting this. I would also note when percentages described as 100% increases are based on small counts.

 

- On page eight it is unclear if “In-person or online courses and workshops.” was meant to be a part of the bulleted list.

 

- It is difficult to follow directly why a focus on information professionals in Latin America leads directly to the inference that “This distribution suggests a higher level of knowledge and awareness among information professionals in Europe compared to their counterparts in Asia and Latin America.” Is this because it is assumed that the former do not need further training? This should be more directly articulated.

 

- Figures 3 and 4/5 have the years and colors swapped for the comparison between 2022 and 2024. I would align these for consistency.

As noted by the authors, the need to rethink the organization and sustainability of supplementary initiatives has become all the more critical as social media platforms have begun to withdraw funding for fact-checking and related efforts to combat online misinformation. The current project serves as helpful scaffolding for policymakers interested in assessing the state of initiatives aimed at improving the resilience of the public to manage the threats posed by online misinformation, with a specific focus on FIMI. While the data limitations (particularly across regions) makes some of the inferences difficult to contextualize, particularly when subset by activity, the insights added by the authors help establish a compelling path forward for stakeholders interested in supporting the ongoing work of fact-check organizations.

If applicable, is the statistical analysis and its interpretation appropriate?

Yes

Is the study design appropriate and is the work technically sound?

Yes

Is the work clearly and accurately presented and does it engage with the current literature?

Yes

Are the conclusions drawn adequately supported by the results?

Yes

Are sufficient details of methods and analysis provided to allow replication by others?

Partly

Are all the source data and materials underlying the results available?

Partly

Reviewer Expertise:

Misinformation, Elections, Social Media, Communications Technologies, Influence Campaigns

I confirm that I have read this submission and believe that I have an appropriate level of expertise to confirm that it is of an acceptable scientific standard.

# Reviewer report: Reviewer response for version 1

**DOI:** 10.21956/openreseurope.21810.r56524

**Published:** 2025-09-10

**Authors:** Laura Teruel Rodriguez

**Affiliations:** 1 University of Málaga, Málaga, Spain

This report analyses media literacy initiatives aimed at countering disinformation, developed by organisations with membership in the International Fact-Checking Network (IFCN). The final sample comprises 73 agencies.

 The study examines two specific periods, 2022 and 2024, in order to observe developments over time. It focuses on literacy activities beyond fact-checking, identifies vulnerable sociodemographic profiles, and explores the funding models of the organisations.

 The theoretical framework is solid, and the justification for this research is clearly presented. A proprietary methodology is developed: *“This tool was specifically designed to identify and categorise training activities in digital and informational skills, based on the information that is publicly available on the websites of the selected organisations.”* This could be further enriched with qualitative data, as discussed later on.

 Initially, the authors state that the sample consists of 88 fact-checking organisations, but in practice only 73 valid records are included ( *“16 local branches of Agence France-Presse (AFP) were excluded due to the lack of specific local information related to them.”*). We find it striking that in the figures the results for 2024 are presented first, followed by those for 2022. It would be more logical to follow the chronological sequence to better reflect the evolution. We would recommend revising this. The dataset itself was not accessible for consultation of the raw data. A more in-depth statistical exploitation of these data could be suggested.

 As regards the results, the report rightly stresses the need for stable funding for fact-checking agencies. They are vulnerable when their financing depends on specific public projects. The proposals put forward to guarantee their sustainability remain somewhat timid. It is useful to highlight the role of the European Union and its commitment to media literacy. A qualitative study is also suggested, through interviews with the heads of these organisations, in order to gain a deeper understanding of their financial reality and the measures they envisage to ensure stability and continuity.

 It is particularly valuable to delineate the audiences targeted by the training activities, as the authors do. They are clearly differentiated by continent, according to sociodemographic needs. However, there seems to be no training specifically aimed at older people; nor does the gender/sex variable appear to be considered in these training initiatives. It would be advisable to address this and to put forward solutions. These findings could be fed back to the organisations so that they take these groups into account. This is especially relevant given that many of these organisations operate with public funding: accountability regarding the results achieved is essential in order to design more appropriate actions for today’s media literacy needs.

 As the authors themselves state: *“No data has been found regarding the number of courses delivered or the number of students enrolled.”* It would be enriching to include whether the training activities are evaluated, so as to identify areas for improvement. This should be proposed to the organisations studied in order to assess their real impact.

 The report is interesting, well-structured, and contains the necessary data to enhance the scope of the digital and media literacy initiatives developed since 2022.

If applicable, is the statistical analysis and its interpretation appropriate?

Yes

Is the study design appropriate and is the work technically sound?

Yes

Is the work clearly and accurately presented and does it engage with the current literature?

Yes

Are the conclusions drawn adequately supported by the results?

Yes

Are sufficient details of methods and analysis provided to allow replication by others?

Partly

Are all the source data and materials underlying the results available?

Partly

Reviewer Expertise:

Disinformation, Fact- Checking, Social Networks, Journalism, Politics, Public Opinion, Polarization,

I confirm that I have read this submission and believe that I have an appropriate level of expertise to confirm that it is of an acceptable scientific standard.
